# Designing a Digital Twin for the Management of Noncommunicable Diseases: Protocol for a Pilot Study and Methodology Validation

**DOI:** 10.2196/75934

**Published:** 2026-02-09

**Authors:** Edgar Ross, Jason Ross, Patricia Beschler, David Guydan, Robert Jamison

**Affiliations:** 1 Atrius Healthcare, 20 Wall Street, Burlington, MA, 01803-4758, United States, 1 2164961571; 2 Department of Anesthesia, Northwestern Memorial Hospital, Chicago, IL, United States; 3 Department of Medicine, University of Basel, Basel, Switzerland; 4 TheraNetrix, Inc., Wellesley, MA, United States; 5 Brigham and Women's Hospital, Harvard Medical School, Boston, MA, United States

**Keywords:** digital twins, NCD, personalized medicine, health risk reduction, biopsychosocial treatment paradigm, noncommunicable diseases

## Abstract

**Background:**

Noncommunicable diseases (NCDs) have become the leading cause of mortality worldwide. NCDs account for 89% of all deaths in the United States and cost the US economy more than US $47 trillion in direct and indirect expenses. NCDs also account for the main cause of disability worldwide, and the incidence is increasing. The leading NCDs include diabetes, cancer, cardiovascular disease, chronic respiratory disease, and mental health conditions. Outside of aging, NCDs are caused by modifiable behavioral risk factors that include smoking, drug and alcohol abuse, unhealthy diet, obesity, and inadequate physical activity, and treatment must be directed to all of these domains. We hypothesize that a digital twin concept can be used to personalize treatment regimens through analysis of data that allows for artificial intelligence–based decision making.

**Objective:**

This study aims to present a methodology to validate this concept, which would provide a new clinical approach toward addressing the leading cause of disability and mortality worldwide today.

**Methods:**

This study will use delta scores between treatment arms to ascertain whether that distribution was normal for each of the study variables. Parametric (eg, analysis of covariance) or nonparametric analyses will be used to examine the variables to determine the impact of digital twin efficacy over normal treatment paradigms.

**Results:**

Recruitment of participants is expected to begin 6 months after study funding has been awarded and the needed approvals have been obtained. The expected results will show that digital twin modeling using the biopsychosocial characteristics of each participant will be statistically significant, supporting using this approach for personalized medical care.

**Conclusions:**

This study can help to identify significant clinical characteristics to help mitigate the impact of NCDs through biopsychosocial treatment paradigms. This paper proposes a statistical framework to evaluate the validity of the platform’s modeling in support of clinical decision making.

## Introduction

### Noncommunicable Diseases

Since the beginning of the 1900s, worldwide life expectancy has more than doubled. Initially, the gains in life expectancy came from improved sanitation and treatment of infectious diseases [1-5]. Further increases in life expectancy are attributed to improved nutrition, neonatal health care, vaccines, and economic growth, resulting in improvements in living standards and coordinated public health efforts [6]. Now the leading cause of mortality and disability in the world is noncommunicable diseases (NCDs). The most common NCDs include cardiovascular diseases, stroke, type II diabetes, certain cancers, and obstructive lung disease. Unfortunately, longer life expectancy that has come from advances in medicine and public health has not been followed by improvement in quality of life because of NCDs [4,5]. Unhealthy behavior and inactivity have led to an increased risk of myriad health-related problems, with increased incidence of dementia, obesity, arthritis, and psychological comorbidities. The incidence of one key risk factor in developing an NCD, obesity, has only gotten worse. This is despite a deeper understanding of its cause and the introduction of new, highly effective drug therapies [7].

Longer life expectancy has led to increased years of disability, and a reduction in quality of life stems from an increase in the incidence of NCDs. These NCDs have led to a substantial increase in health care costs and economic and public health burden [8]. NCDs are lifestyle diseases from deeply ingrained behaviors that can be difficult to change. Modifiable risk factors include poor diet and nutrition, obesity, alcohol abuse, cigarette smoking, lack of regular exercise, and environmental and socioeconomic factors [9-12]. Although a few NCDs account for the highest impact on public health and have many commonalities and similar treatments, the prevention and management of these NCDs on the individual level is actually very complex. This complexity stems from the differences in genetics, backgrounds, resources, access, and attitudes, and when taken together, have been labeled biopsychosocial medicine [13,14]. Personalized medicine applies evidence-based treatment guidelines in a framework that factors individual patients’ differences, making treatment success more likely. Biopsychosocial medicine recognizes genetic predispositions as risk factors for the development of NCDs, but even these risks can be overcome by behavior change and effective treatments [15].

NCDs are a general category that has been categorized into different diagnoses. The individual health risks of these NCD diagnoses can vary dramatically. The initial diagnosis of an NCD requires an assessment of risk and treatment recommendations that can vary from self-management to the prescribing of medications or even urgent interventions. Although treatment can vary, lifestyle modification should still be part of a comprehensive approach that should include an understanding of the biopsychosocial background that is unique to the patient. Ideally, active management of NCDs begins with prevention through lifestyle modification. Self-directed management using a biopsychosocial approach includes personal recognition of concerning trends that lead to increased health risks. Both preventative primary care and societal public health initiatives are part of this response [16-18]. Once the presence of an NCD has been diagnosed, treatment options usually begin with pharmacological treatment. If an NCD is recognized late, urgent care could be required; this can include procedures or even surgery [19]. All evidence-based guidelines include an emphasis on lifestyle modifications along with needed interventions. Beyond the acute stage, effective treatment and health risk reduction for these chronic conditions require a therapeutic alliance between the primary caregiver and the patient and an appreciation of the biopsychosocial background of the patient. Personalized medicine has been advocated to develop and support this alliance [20-22].

### Treatment of NCDs

The treatment of NCDs is guided by the consensus guidelines developed by international and national public health agencies and specialty groups through the analysis of existing literature and expert opinion. These diagnostic-specific guidelines have been validated through randomized controlled trials (RCTs), comparison studies, meta-analysis consensus meetings, and authoritative expert opinions [23-28]. Although guidelines are very helpful in standardizing evidence-based treatment, their application and adoption by a patient can be limited because of differences in biopsychosocial backgrounds. The goal of personalized medicine is to consider these differences and tailor treatment strategies for optimal outcomes. An example of this complexity is illustrated by the barriers to overcoming physical inactivity.

The cause of NCDs is multifactorial, but one of the most important risk factors for developing an NCD is physical inactivity. Physical inactivity is associated with obesity and developmental musculoskeletal pain. The comorbidities of chronic pain can make the restoration of a healthy lifestyle much more difficult [29]. Yet, the health benefits of sustained activity can be evident within 4 weeks, the largest benefit seen among people with the most sedentary lifestyles [30,31]. A goal of 150 minutes of moderate aerobic activity per week has been shown to be the amount that provides the most benefit. This is information that has been well documented since the early 1950s. Most recently, inadequate exercise has even been associated with an elevated cancer risk [32]. Yet nearly one-third of all adults worldwide do not reach recommended weekly activity levels. Most medical treatments do not include recommendations for needed behavioral changes to reduce the risks of increased NCDs. When behavior change recommendations are made, only a small minority of patients are self-motivated enough to follow through with provider recommendations. The vast majority of patients are not sufficiently motivated to make lasting changes in behavior that could significantly improve their health status when recommendations alone are made. This includes increasing and maintaining higher activity levels [33]. Certain psychological traits, such as catastrophizing when combined with chronic pain, are an even more difficult barrier to compliance. Catastrophizing is known to respond to psychological therapy [34]. Other psychological comorbidities, such as depression and anxiety, can also impact compliance with treatment recommendations [35,36]. These examples illustrate the reason a clinician needs to have a holistic perspective on their patients.

### Patient Tracking

Tracking a patient’s progress with a chronic condition is key to providing feedback to the patient about their progress and supporting treatment decision-making. Examples of tracking patient progress include objective measures such as laboratory tests, serial blood pressures, activity levels, diet, and respiratory reserve, along with regular subjective measures of patient well-being; all are useful longitudinal measures that can be used to measure compliance, identify barriers that are impeding progress, and personalize care. The data collected should also be continuously screened for acute changes that suggest concerning health risks that require urgent reevaluation. The data collected can be used for disease-specific risk assessment calculators to track treatment effectiveness and longitudinal progress [37-39]. Successful treatment of NCDs often requires lifestyle changes that are deeply ingrained. Contextual and regular feedback with personalized messaging is significantly more effective in reinforcing beneficial behaviors than periodic brief conversations typically associated with health care appointments [40,41]. Using a personalized medicine approach that is facilitated by a DT specifically developed for NCDs has the potential to significantly improve treatment efficiency, outcomes, and decrease the enormous public health burden that NCDs cause. Constructing a DT requires a system that combines multiple different technologies and a methodology that facilitates adoption by a clinician and their patients. A DT is able to combine the biopsychosocial background of a patient, apply the relevant literature, provide decision support, and continuously adapt to a changing clinical picture. A personalized DT will be constructed using the unique characteristics of a patient’s biopsychosocial background, risk of presenting with an NCD, and will use the relevant evidence-based literature that contains large numbers of participants to develop a personalized treatment plan for the N-of-1 patient.

### Adopting the DT Concept to Treating NCDs

Since the introduction of DTs, the management of increasingly more complex manufacturing processes has evolved from a product focused on the entire process, including design of the product, building, use, and disposal, which collectively is known as product lifecycle management (PLM). The concept of PLM has been pivotal in rethinking manufacturing from a product focused on the management of the entire life cycle [42]. The key advantage of this approach is the ability to simulate performance, identify, and optimize key variables through continuous measurement and the evaluation throughout the lifecycle of a product. Health care has a similar PLM concept known as an episode of care (EOC). This concept is well-established and has been used to profile and improve care and support cost containment programs [43,44]. EOC begins with the evaluation and assessment of a patient, the development of an active treatment plan, followed by tracking of outcomes when a treatment goal has been reached. Risk adjustment measures have been added to EOC management to better understand reasons for treatment variability and differences in clinical efficiency and outcomes [45,46]. These risk adjustment measures typically include physical, psychological, and demographic backgrounds of patients [47]. Medical care has frequently looked to industry and manufacturing as a model for clinical process redesign [48]. However, evaluation and redesign of clinical processes and pathways use EOC data that are retrospective and aggregated data to optimize processes [49,50]. The similarities between PLM and EOC are noted in Table 1 below.

**Table 1.** PLM^a^-EOC^b^ comparison.

Stages	Description
PLM stage	
Create	Evaluate a need, develop, and design a product specifically for an opportunity
Build	Build prototype, needed manufacturing infrastructure, and supply chain
Use or sustain	Product behavior, performance, prognostics, service, and repair
Dispose	Project archiving, regulatory compliance, and completion of product life cycle
EOC stage	
Evaluation and assessment	Comprehensive patient evaluation, including biopsychosocial profile
Active treatment	Comprehensive treatment plan formulated and implemented
Follow-up	Track treatment plan progress with revisions as indicated
Treatment course completed and outcomes assessment	Patient tracking, change in health risks, and process efficiency

a PLM: product lifecycle management.

b EOC: episode of care.

### Elements Needed to Construct a Personalized NCD DT Program

**Figure 1.**
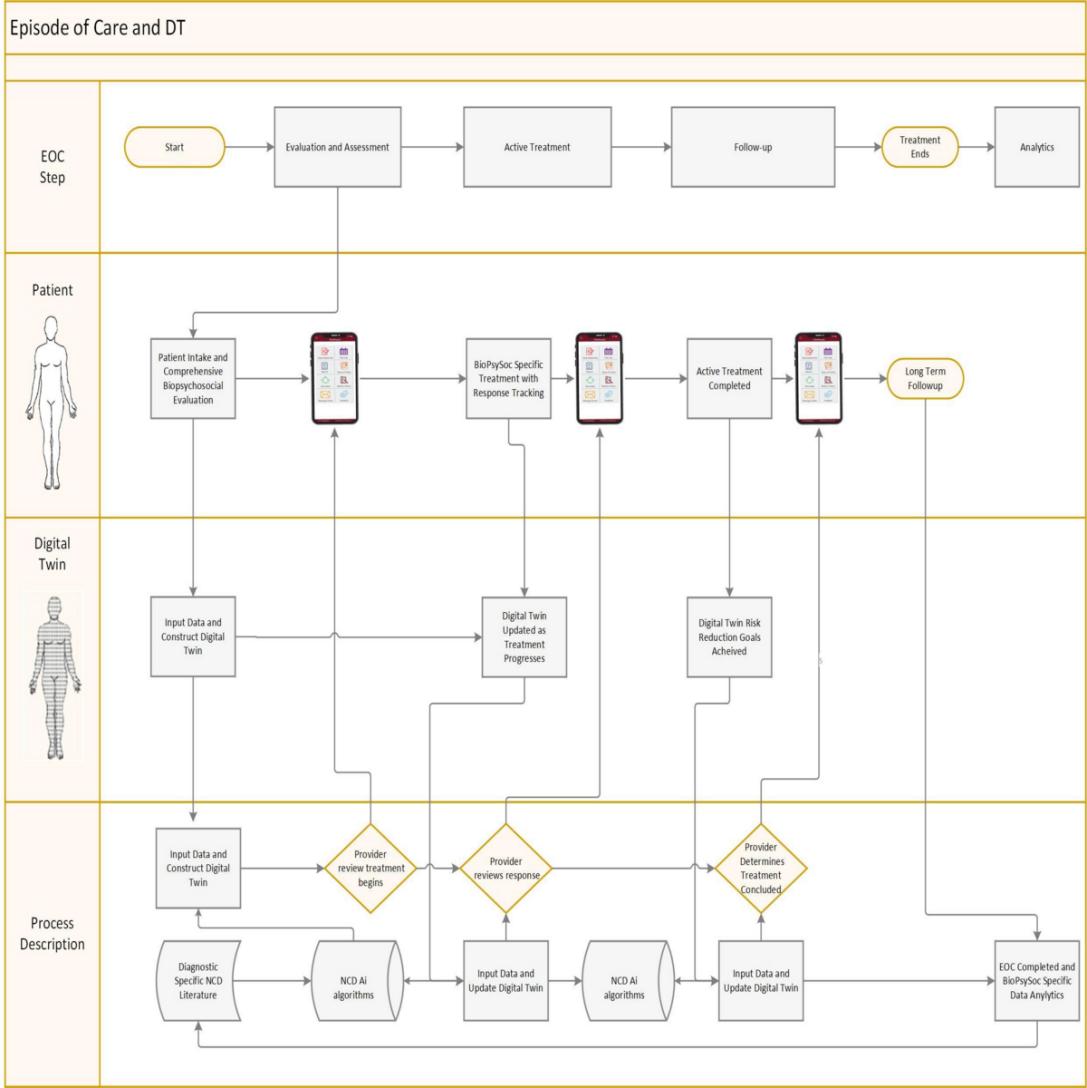
Integrating a digital twin into the episode-of-care structure. AI: artificial intelligence; BioPsySoc: biopsychosocial; EOC: episode of care; NCD: noncommunicable disease.

The process of incorporating a DT to manage a PLM could also be used to manage an EOC. A DT of a patient would be developed with historical and real-time patient data, diagnosis-specific risk assessment using simulation, machine learning, computational modeling, and large language modeling (LLM) review of the relevant evidence-based literature to obtain a unique biopsychosocial profile of the patient. Instead of the typical health care process that consists of intermittent patient-provider contact, the DT would be continuously updated by bidirectional data streams between the actual patient and their personal simulation. At the end of the EOC, the analytics collected would be used to improve and enhance the treatment algorithms that can be used to further clinical efficiency and improve outcomes. Figure 1 illustrates how a DT can be used to manage an EOC.

A comprehensive structure that can be used to develop a personalized DT that has the ability to assess and provide decision support in health care requires the following elements:

1. Evidence-based literature is appropriately structured that will inform the background for algorithm decision-making that assesses health risk and provides treatment options for patients with varied backgrounds.
2. The actual patient is evaluated using a biopsychosocial analytic protocol.
3. The virtual patient is constructed as a synthesis of the results of actual patient evaluation and evidence-based literature-based artificial intelligence (AI) algorithms.
4. Secure continuous 2-way data connection between an actual patient and their DT.
5. Data supported DT recalibration through the active treatment phase of the EOC.
6. Tracking treatment outcomes, data analytics and integration, modeling, and refinement of treatment decision support and effectiveness.

The European Virtual Human Twin Initiative (EDITH) project was a 2-year project sponsored by the European Commission to develop a roadmap toward the development of a virtual human twin. In what has been referred to as a moonshot, the EDITH creates the infrastructure and processes with a goal of describing the entire known human pathophysiology [51]. To complete this project, immense data sets, computing power, understanding of both normal and abnormal physiology, and the mechanisms of interventions are needed. This scope of work will require considerable development in many areas that puts it out of reach because of the numerous technical limitations and overall cost. Narrowing the focus of a DT to certain diseases that have smaller numbers of variables and treatment pathways has been used successfully to overcome these technical limitations. The EDITH project’s report “Vision for the VHT and Roadmap Outline” categorizes DTs into three categories. They include (1) generic DT: the expected accuracy is within the range of the studied population, (2) population-specific DT: the expected accuracy is sufficiently close to a mean or median range of values of the referenced population, and (3) participant-specific DT: the accuracy is close to each individual in the reference population with similar characteristics.

Although the above 3 categories can be viewed as distinctive and separate, the categories could also be viewed as a series of stages in a development pathway toward the goal of a DT that will be able to deliver personalized medicine and early disease detection to an N-of-1 (Table 2). All 3 of these stages represent initial development approaches. It is expected that ongoing comprehensive outcome data collection, including sensor data, providing sufficient experience for statistical analysis, will improve the predictive modeling of DTs.

**Table 2.** Overview of digital twin development options.

DT^a^ type/stage	Input data	Attributes/ d eficiencies	Required data	Clinical decision support
Generic	Studied populations, accuracy within bounds of referenced population	Least number of variables, easier to develop, may not accurately reflect individuals with important differences outside bounds of referenced studies	Large data sets with similar methodologies, outcomes, and diverse biopsychosocial backgrounds	Generalizable DTs might not be accurate for unstudied key individual differences
Population-specific	Relevant participant-specific study means, medians, or equivalent	Larger number of variables, improved modeling, may not accurately reflect individuals outside SDs	Need studies with sufficient data detail, and subgroup analysis for negative studies	Improved virtual DT representation for patients who fit clinical trial profiles
Participant-specific	Sufficient individual treatment goal–specific accuracy	Largest number of variables, significant evidence-based data mining for AI^b^, and in silico modeling will improve DT accuracy	Comprehensive literature review and analysis to find connections that could impact patient care	Most accurate DT, with ability to predict barriers that can impede reaching treatment goals

a DT: digital twin.

b AI: artificial intelligence.

### Medical Literature and Evidence-Based Medicine

Access to large data sets is key to training the AI algorithms needed to deliver the promise of DTs and personalized medicine. Data sets needed for training include well-designed clinical trials for decision support, risk assessment tools that can provide feedback on treatment interventions and large observational cohorts for phenotyping of diverse populations, and prediction of response to interventions. The “All of Us” research program funded by the National Institutes of Health is one example of a data set that could be used to develop a DT infrastructure. With a stated goal of collecting data from a million highly diverse groups of participants, the project will provide considerable biopsychosocial data that advance the goal of personalized medicine [52-54].

The last few decades have seen rapid advances in understanding the pathophysiology of the majority of diseases and the development of new treatment options. The “bench to bedside” process has relied on RCTs of varying designs to obtain needed approval for any recommended clinical treatment. RCTs typically rely on the recruitment of homogenous populations of potential responders to determine the efficacy of a medication or an intervention. RCTs have become the gold standard for evidence-based medicine; however, clinicians treat heterogenous patient populations who often do not share the characteristics of participants found in RCTs. Diseases do not often present with identical symptoms from one patient to another. Treatment response and side effects can also vary greatly from one person to another. Because of this, personalized medicine has been embraced as a way to improve the care of the individual patient. This approach seeks to tailor treatment to each person to account for genetic, physiological, biochemical, behavioral, and environmental differences [24,55]. To be most effective, personalized medicine requires the consideration of a large number of variables. The results from traditional RCTs do not easily lend themselves to providing the needed information to practice evidence-based personalized medicine [17,56].

A consensus panel review of clinical trial design for pain (see the study by Edwards et al [19]) made suggestions to expand and modify existing methodologies to incorporate the needed information that allows for a more accurate understanding of how individual factors predict and influence clinical treatment decision-making. Although the Initiative on Methods, Measurement, and Pain Assessment in Clinical Trials panel recommendations are specific to pain management studies, their recommendations also have general application for other conditions [57]. However, for the busy clinician, implementing personalized medicine can become very complicated and time-consuming and not easily adapted for the average provider without support.

Although AI in medicine has received considerable attention because of its promise to improve health care, its true potential in clinical practice has not yet been realized. AI is a broad term that refers to computer-based algorithms that can mirror tasks and abilities of human intelligence. There are many different forms of AI, but all share a common goal—using computer-based algorithms that review data to uncover diagnoses, assist clinical decision-making, and uncover new therapies [58,59].

AI possesses advantages over human intelligence through its ability to assimilate vast amounts of data, to assist with mundane repetitive tasks, and to consider numerous variables at once to assist with decision-making. This process exceeds the human capability to assimilate information and can lead to greater consistency and speed in decision-making, to remove bias based on anecdotal experience, and to quickly incorporate new advanced treatments [60,61]. The validity of evidence-based literature that would be used by AI decision-making carries with it a key risk factor, the underrepresentation of minorities and certain demographic groups [62-64].

The disadvantages of AI include biased algorithms introduced by racial, gender, socioeconomic, and age prejudice found in the medical literature. Patient safety, ethical, and privacy concerns covering data collection can be ignored. Algorithms can also lack transparency, compromise clinical implementation, and cause distance between the health care provider and the patient [65]. Appropriate governance and transparency of AI are needed to ensure that bias is not introduced into clinical decision-making. Recommendations made by the DT program will be continuously monitored, and clinician feedback on decision-making will be reviewed and used to ascertain that DT algorithmic decision-making is appropriate and effective. Adjustments when needed will be transmitted to the study personnel and clinicians.

The most compelling use of AI in medicine has been in the role of supporting resources that a clinician can easily and effectively use in daily practice. Data supporting this type of collaboration have the potential to offer compelling synergism [66]. The combination of clinician support and AI-enhanced clinical care could greatly improve the goal of implementing personalized medicine [67].

### Health Care and DTs

A DT is a virtual representation of a physical object that is used to simulate a process. This simulation can be used to predict the impact of a new process or to modify an existing process to improve risk identification and clinical efficiency. It has the potential to contribute to real-time decision-making to effectively predict outcomes and to make cost-reduction improvements. Because of these attributes, a DT with virtual representations has been shown to have widespread use in industry to simulate the impact of proposed modifications and run “what-if” scenarios before implementation. When a modification is introduced, DTs can be continuously updated with performance data that can be used to manage the process in real time. This real-time monitoring in parallel with the updating of a DT leads to improved management and system stability while enhancing system security [68,69].

### Health Care Simulation

Simulation in health care has long been used in training and in improving outcomes in areas such as diagnosing cardiorespiratory arrest, designing mental health treatments, and, most recently, planning treatment interventions such as surgery. The major drawback of simulations is that they are fixed; they do not receive new data, and they are generally not easily modifiable when clinical situations change [70-72]. DTs differ significantly from simulations because of the continuous flow of data from the patient to the DT, which can lead to modification of the DT in real time by simulating the patient’s clinical course [73]. When used in health care, a DT is a virtual representation of a patient. A health care DT has essentially the same attributes as an industrial DT. A patient DT is constructed by analyzing relevant characteristics and problems of that individual through AI to simulate the potential treatment options and provide the best clinical course. DTs have the potential to improve treatment compliance, address individual risks, reduce barriers to improvement, intervene before side effects become problematic, and enhance a therapeutic alliance between the patient and provider. This approach of combining individual characteristics and AI data from a DT that is unique to a patient helps to support the goal of personalized medicine. The health care DT represents a key technology that brings together the advantages of a comprehensive picture of a patient, leading to a precise diagnosis and a personalized treatment plan that is evidence-based yet still personalized to that individual [74,75]. As treatment progresses, the DT is continuously updated by monitoring the patient’s progress through data collection and providing feedback to the patient and treating provider. The changing clinical picture leads to a modification of that patient’s DT.

### Treatment Trajectory

**Figure 2.**
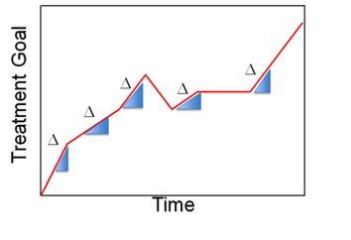
Hypothetical treatment course.

Treatment trajectories leading to clinical improvement rarely follow straight linear paths toward a goal [76]. Figure 2 illustrates this for a hypothetical patient. As noted in the figure, the delta of change over a course of treatment can vary. These changes could lead to different approaches or consideration of different treatment options. Ongoing tracking data from the patient and revision of the patient’s DT, as these data become available, modify the DT and can help identify these deltas. This process can provide new insights and a change in clinical decision-making. This back-and-forth data exchange in real time between the patient and that person’s DT can be key to providing personalized medicine.

### Designing and Developing a DT for NCDs

To our knowledge, a protocol designed to assess the use of a DT to treat NCDs has not been conducted or reported in scientific literature. As noted above, NCDs are lifestyle diseases that can require a multidisciplinary approach toward effective diagnosis and behavior change to impact the long-term reversal of health risk factors using a biopsychosocial approach. The interdisciplinary approach needed to effectively treat a patient with an NCD requires the following:

1. Comprehensive medical review and treatment of the consequences of the NCD.
2. Biopsychosocial evaluation with risk assessment that can impact treatment goals.
3. Psychological assessment and comprehensive treatment when needed.
4. Modification of behaviors that have led to the development of the NCD.
5. Evaluation of current activity, presence, and/or treatment of pain, and integrated rehabilitation when identified.

The design and implementation of a DT for an NCD is as follows:

1. Conduct a literature review of appropriately designed studies related to any particular NCD. These studies will provide an analysis of health risks and therapeutic clinical trials that include both health risks and responder analyses. This information will be used through supportive AI decision-making to form a DT and to offer clinical support.
2. Obtain a patient-centered interface that connects with an electronic medical record to collect existing data. This will allow for the assessment of the patient’s baseline key health indicators, to track key variables to be used to update the patient’s DT while providing feedback, including contextually specific motivational messaging, and to support the provider. Identification of patient engagement issues and possible measures of treatment outcomes would be generated. Tracking of key patient measures will be facilitated by peripherals enabled to gather objective data such as blood glucose, blood pressure, daily activity, oximetry, and weight [77].
3. The computational process will begin and continue throughout the study process as data are collected, and the patient status is defined by analyzing key health data to form both the initial and updated individual DT using a biopsychosocial framework.
4. Collect deidentified data that will further refine the algorithms used to create the DT and future DTs.

The NCDs that account for the most common cause of death in the United States are cardiovascular disease, cancer, chronic respiratory diseases, and type II diabetes [78]. All of these NCDs are directly influenced by maladaptive behavioral lifestyles such as poor diet, insufficient levels of activity, cigarette smoking, metabolic disorders, and medical conditions, including hypertension and hypercholesterolemia [24]. Biopsychosocial risk factors for the NCDs can include the following items listed in Textbox 1.

Textbox 1. **Biopsychosocial risk factors for noncommunicable diseases.**

- **Genetics**

- Metabolic risk factors

- **Behavior**

- Smoking
- Excessive alcohol intake
- Unhealthy diet
- Low levels of activity

- **Environment**

- Air pollution
- Exposure to carcinogens and toxic wastes
- Temperature extremes
- Civic infrastructure

- **Demographics**

- Access to health care
- Income
- Age/gender
- Ethnicity

### Barriers to Progress and DT Treatment Planning

Effective treatment requires an understanding of barriers to goals, including environmental and demographic factors. Genetics is known to play a role in NCDs, but with personal feedback and targeted treatment, the genetic impact can be mitigated.

**Figure 3.**
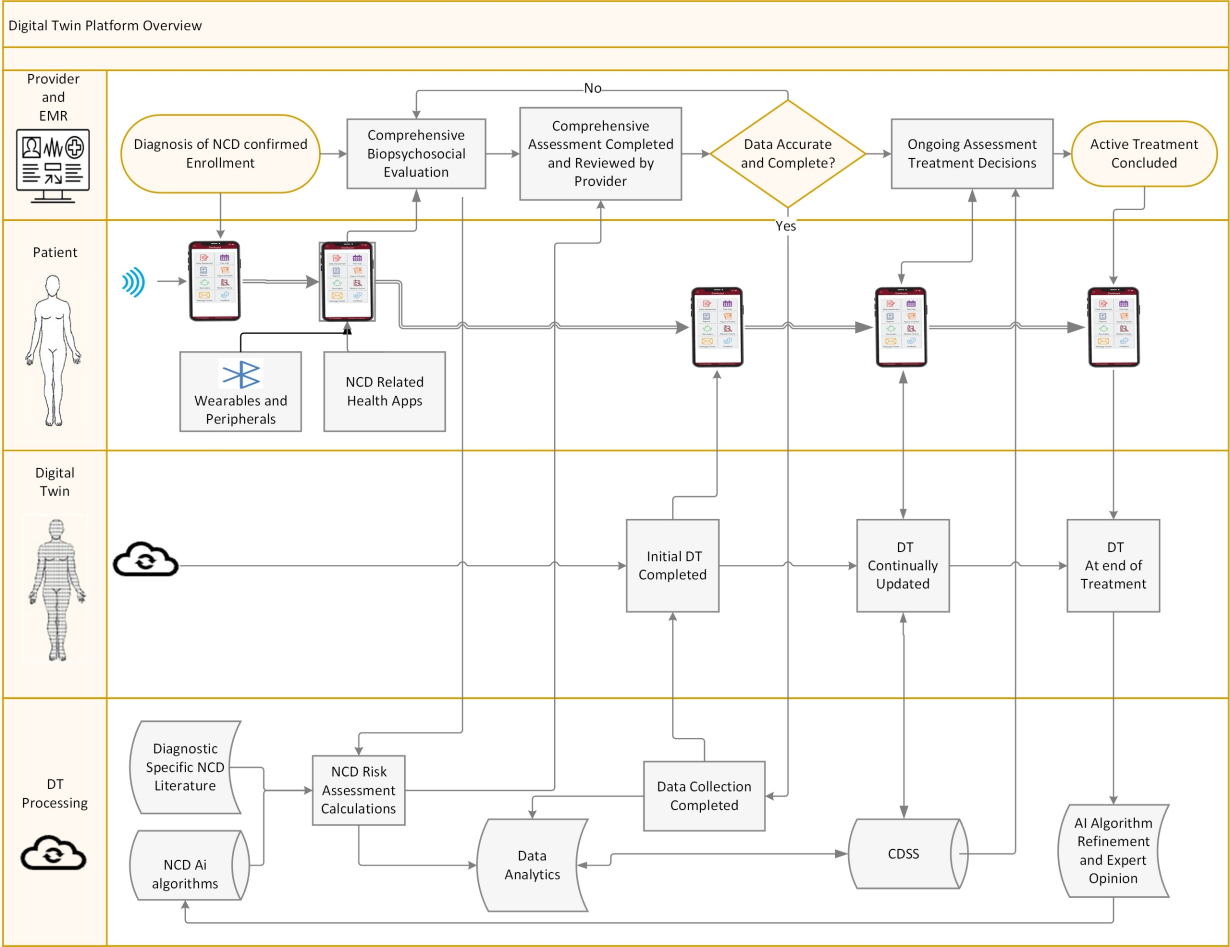
Digital twin overview. DT: digital twin.

**Figure 4.**
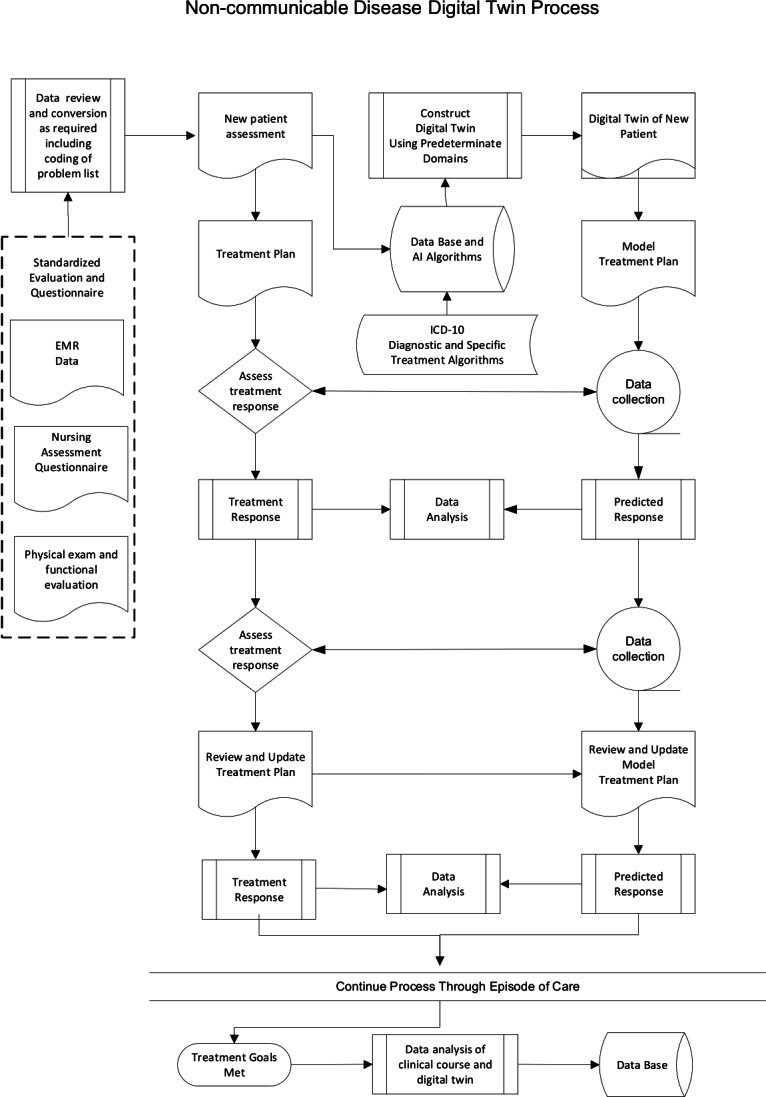
Overview of inputs and information flow needed to construct a DT for NCDs. AI: artificial intelligence; EMR: electronic medical record; *ICD-10*: *International Statistical Classification of Diseases, Tenth Revision*; NCD: noncommunicable disease.

We have previously reported on our patient engagement platform that has been used in detecting and treating risk factors for NCDs, including diabetes and chronic pain (MobileNetrix) [79-81,82]. We have also reported on the ability of our patient engagement platform to identify psychological traits that can impact compliance with treatment recommendations and comprehensive care [83]. The patient-facing app is customizable for conditions being studied and is easily adapted to the treatment of all types of NCDs. The app is designed to reinforce patient engagement with their provider, as well as promote behavioral change through automated motivational messaging and psychological principles of change. It can also track patient progress and easily incorporate peripherals and wearables that will provide the needed data to construct a patient-specific DT [84,85]. The programming flowchart for an NCD DT is illustrated in Figure 3. A more detailed explanation of each element is presented in Figure 4.

Biological systems are nonlinear. As noted above, the clinical course of an individual is also usually nonlinear, characterized by many deltas in an EOC toward achieving a treatment goal. Any DT must allow for this variability and continually track the individual through efficient data collection and updating of the DT. Our model for a DT will have 4 essential components. These four components will include (1) the identified patient, (2) the unique model of the patient or DT, (3) rapid and timely synchronized data exchange between the individual and the DT, and (4) ongoing analysis of potential variables that are predictive at a macro level. Using a back-and-forth process and a large number of variables, updates of DTs can include an analysis of all the original variables, including their potential relationships and impact. The DT platform consists of the use of the nearly ubiquitous smartphone that will provide patient-facing data collection and biometric and health data from wearables (eg, Google Fit and Apple Health) and a communication portal with 2-way messaging. A DT Health Management App for both iOS and Android platforms that will be HIPAA (Health Insurance Portability and Accountability Act)-compliant will offer electronic medical record integration, enhanced data entry features, Bluetooth-enabled device syncing (eg, glucose monitors), and emergency alerts. This app will be designed to be robust, scalable, and secure, ensuring seamless integration of various components to deliver a comprehensive health care solution. A custom application programming interface (API) will facilitate secure and efficient data exchange between the mobile app, backend infrastructure, and web portals. An administrative portal will be used by the treating provider to access data with electronic medical record integration and offer decision support, and a super-admin portal will monitor system performance and the computational analysis that supports DT construction and updates. A collaborative information model has been proposed that will be able to evaluate complex systems with multiple inputs interacting together to define a specific outcome [45]. When data to construct or update a DT is incomplete, expert knowledge–based evidence will be used. Figure 3 details DT functioning and data processing, flow, and provider supervision. Table S1 in Multimedia Appendix 1 illustrates communication processes and their APIs.

### Study Objectives

The goal of this study and the primary objective is validation of our automated patient engagement programs to guide participants toward activity gains, sustainability, opinions of the situational relevance of the engagement tools, and other key performance indicators using a DT. The research plan has several objectives. It will examine the impact that personalized health care messaging and treatment recommendations can have on individuals. Secondary objectives include (1) testing published guidelines on a heterogeneous population, (2) developing a methodology that combines multiple different biopsychosocial characteristics into a personalized treatment plan that improves on and is responsive to an individual’s unique background, and (3) refining algorithms that support clinical decision-making of a clinician.

## Methods

### Validation Methodology for a DT NCD

This proposed study will use the smartphone (iOS and Android) and MobileNetrix as the health management systems platform for a patient DT NCD study. A HIPAA-compliant DT Health Management App will be created, supported by AI and computational analysis. The administrative portal will automatically poll each participant after being identified as a candidate based on validated criteria of having one of the NCDs studied in this protocol. The 3 NCDs that will be included are (1) cardiovascular disease, (2) type II diabetes (non–insulin dependent), and (3) chronic obstructive pulmonary disease.

Candidates will be screened through a 2-step evaluation process. After informed consent is received, the participants will be invited to download the study app. Following a successful app enrollment, a screening questionnaire specific to the provisional diagnosis will be sent through the study app that will contain a link to a designated questionnaire. Once complete, if the individual is found to qualify for the study through the screening questionnaire, an appointment will be made for a comprehensive evaluation to confirm the diagnosis and related risk factors. Table 3 details the screening process for each NCD.

**Table 3.** Screening process for specific noncommunicable diseases.

NCD^a^ diagnosis	Screening questionnaire	Confirmatory evaluation
Cardiovascular disease	AHA PREVENT^b^ risk calculator [86]	Laboratory and physical exam: diabetes (%), current smoking (%), mean total cholesterol (mg/dL), mean systolic blood pressure (mm Hg) [87]
Type II diabetes	Diabetes Risk Score Questionnaire [88]	Fasting blood glucose >126 mg/dL, 2-hour OTT^c^ blood glucose >200 mg/dL, HbA_1C_^d^>6.5% [89,90]
COPD^e^	CDQ^f^ questionnaire [91]	Postbronchodilator spirometry ratio of FEV_1_/FVC^g^ of less than 0.70 confirms the presence of persistent airway obstruction and COPD [92]

a Noncommunicable disease.

b American Heart Association Predicting Risk of Cardiovascular Disease Events

c Oral tolerance test.

d Hemoglobin A1C.

e Chronic obstructive pulmonary disease.

f COPD diagnostic questionnaire.

g FEV_1_/FVC: forced expiratory volume in 1 second to forced vital capacity.

If the risk factors are confirmed, an additional evaluation will be used to determine other specific risk factors associated with that condition, including level of physical activity, psychological traits or maladaptive behaviors, and barriers to improvement, including the presence of pain, sleep disruption, smoking, and other comorbidities related to worsening health. For an overview of these inputs, refer to Table S2 in Multimedia Appendix 1. These secondary domains will become the individual’s study-specific targets. All of these factors will be incorporated into the participant’s DT. The DT will be shared with the patient through the study app and reviewed with the patient during an in-person follow-up visit. The study platform will provide daily questionnaires that are specific to each NCD category and problem list. Objective tracking of the patient’s activity level and physiological measures (such as blood pressure, glucose levels, and pulse oximetry) will continue throughout the treatment period. All these daily data points will be used to update the DT and will appear in the participant’s study portal that is part of the patient-facing app. The updated DT primary endpoint will reflect the change in health risk. Participants found not to be compliant with the program will be contacted and interviewed regarding barriers and their impressions of the program. Clinician decision-making will also be tracked. Variance from DT treatment recommendations and the impact on NCD risk factors will be reviewed and statistically compared to expected outcomes from relevant evidence-based literature.

Automated contextual daily motivational messaging that aligns with the participant’s behavioral traits will continue throughout the study period. Daily compliance reminders for medication regimens will be incorporated into the study app. The program goal will be a 37% reduction in long-term health risk from baseline as determined by the NCD-specific risk questionnaire (Tables S1 in Multimedia Appendix 1). This is considered to be a meaningful reduction in health risk using the financial modeling included in this reference that will sustain a DT program [93]. The number of participants in each arm reaching this goal will be used to evaluate the primary objective of this study as noted above. Each candidate will be followed for 6 months, and the study length will be determined by enrollment rate.

The program’s daily questionnaire options include (1) the participant’s impression of the program and global impressions of change, (2) Global Activity Limitation Index, (3) Brief Pain Inventory, (4) medication adherence and compliance, (5) daily diet summary, (6) quality of sleep, and (7) smoking and alcohol consumption.

### Validation Study Material and Methods

#### Overview

All eligible participants will be provided sufficient details on the study so that they can provide informed consent prior to enrollment. This process will include a complete discussion of the study’s purpose, the study procedures, and the risks and benefits of participation. The risk discussion will include the risks and benefits of the use of AI for health care. All study data collected will be kept confidential and securely stored. All personal identification will be removed from the study data. Personal identifiers will be available to treating providers. Participation in this study is completely voluntary, and participants can withdraw at any time. The decision to participate in this study will not affect access to care. All treatment decisions will be made by the patient’s provider only. This study will conform to all international standards covering human subjects for this research project.

This study will be approved through the Institutional Review Board (IRB) of Brigham and Women’s Hospital in Boston and registered on ClinicalTrials.gov. The health care providers participating in this validation study will identify and sequentially include sufficient patients to validate this process. An effort will be made to include a diverse group of enrollees to allow for a subgroup analysis of the impact on decision-making in the biopsychosocial and personalized medicine. The potential participants will be new patients in their practice identified as having one of the NCDs that are being targeted in this study. The potential participants will be screened for both inclusion and exclusion criteria listed below. If found to be eligible, the personal smartphone of the identified participants will be polled using SMS text messages to determine interest. Interested individuals will be provided the study app and questionnaires associated with their provisional diagnosis. If the screening questionnaires are compatible with an NCD diagnosis, secondary questionnaires will be transmitted through the study app and an appointment will be made for confirmation of the diagnosis using the criteria listed in Textbox 2. After the confirmation of the diagnosis, each participant will be evaluated by a multidisciplinary team that will include the treating provider, a psychologist, and a physical therapist. After informed consent and sufficient instruction, the participants will be formally enrolled and treatment will begin. Relevant outcomes such as improvement in activity duration and frequency, as well as response to automated messaging, will be tabulated. Figures 4 and 5 illustrate the clinical pathways that will be implemented in this clinical trial of an NCD-focused DT, along with the feedback loops that will be used to refine the validity and increased accuracy of the DT toward being able to support a biopsychosocial personalized medicine at the level of N-of-1. The eligibility criteria are listed in Textbox 2.

Textbox 2. **Inclusion and exclusion criteria.**

**Inclusion criteria**

- Personal smartphone (either Android or iPhone iOS).
- Clearance from the primary care provider.
- At-risk noncommunicable disease and confirmed by in-person evaluation.
- Willingness to participate.
- No contraindications to participating in treatment recommendations.

**Exclusion criteria**

- Diagnosis of stage IV cancer or any other advanced malignant disease.
- Acute osteomyelitis or acute bone disease.
- Present or past DSM-5 (*Diagnostic and Statistical Manual of Mental Disorders* [Fifth Edition]) diagnosis of schizophrenia, delusional disorder, psychotic disorder, or dissociative disorder that would be judged to interfere with study participation.
- Pregnancy.
- Any clinically unstable systemic illness judged to interfere with treatment.
- Any condition requiring urgent surgery or a procedure.
- An active substance use disorder, such as cocaine or IV heroin use that would interfere with study participation.

#### Study Risks

The risks of participation in this study are minimal, mainly because the clinician will make all the treatment decisions. All treatment that the participants receive will be standard-of-care treatment that any patient with the same diagnosis would receive. Although the risk is low, the participants’ data and communications could be breached. As both platforms are online, the risk of unauthorized viewing or use of online data is higher than that of physical records. The entire platform will be hosted within a HIPAA-compliant firewall, and the smartphone patient-facing app will be compliant with all relevant standards to maintain patient confidentiality. The MobileNetrix app has been used previously in clinical trials conducted at Mass General Brigham and is Veracode tested. Finally, participants may become uncomfortable and emotional while completing some of the questionnaires. Risks of AI include unrecognized biases and hallucinations, leading to the potential of erroneous decision support recommendations.

#### Enrollment Procedures

The smartphone will be used to communicate with the eligible participants. They will be sent a poll using an SMS text messaging–based survey app for eligibility for in-person evaluation. The administrative portal will automatically categorize participants into a defined level of risk of their NCD. The participants with confirmed NCDs will be evaluated by the interdisciplinary team noted above, and a personal treatment regimen will be designed for them to reduce their health risks, along with a DT that reflects their state of health at enrollment. There will also be a comparison-matched group of participants who will be randomized to a treatment-as-usual condition, where they will not have access to the DT technology. After sufficient instruction, the participants in the experimental treatment condition will be tracked daily and be provided with reinforcing messaging and automated problem-solving based on their DT. Any changes in their health-related behaviors will automatically be reflected in an updated DT. Efficacy of the DT and personalized medicine approach will be measured by the number of individuals reaching a 37% risk reduction, with comparison between enhanced care using the DT and treatment as usual (TAU). TAU is defined as the treatment of one of the study’s NCDs using published guidelines and literature as a guide, but without using any of the DT study interventions. Secondary outcome measures that will be tracked will include each of the diagnosed NCD risk factors and other outcome measures, including compliance and helpfulness ratings of the participants and treating providers. In addition to studying the 3 NCDs noted above, we will select patients diagnosed with type II diabetes with pain for further in-depth analysis, as detailed in the Statistical Methods section.

#### Statistical Methods

We expect that both groups (DT Experimental and TAU Control) will have missing data. We will assess whether missing data are more prevalent in one group or the other, and if there are differences among those who drop out early and those who complete the 6-month trial. We will also use corrections for multiple comparisons to reduce the likelihood of type I error (Benjamini-Hochberg correction). A calculation will be produced to determine whether the significance value to interpret statistical significance needs to be different from the standard *P<*.05. Power calculations, as outlined by Cohen [94], will be performed to determine the probability of detecting clinically significant differences between treatment conditions in the primary area of measurement. These calculations will assume a 2-tailed test and α level of .05 that use of a DT will improve activity interference on the activity subscale of the Brief Pain Inventory. The Brief Pain Inventory was chosen as a primary outcome measure. This is because persistent pain and other symptoms are often associated with NCDs that can adversely affect quality of life. The Brief Pain Inventory includes 7 items that assess the degree to which pain and other symptoms interfere with daily living and activity, including general activity, walking ability, normal work (both outside the home and housework), relations with others, sleep, mood, and enjoyment in life (0=does not interfere; 10=completely interferes). These items can be collapsed and averaged into one measure of interference.

In our previous work [85,95-97], we had a high rate of consent to participate among recruited patients, with minimal attrition. We assessed power based on our preliminary data and previously published studies. The power analysis revealed that a sample size of 100 participants (50 per treatment arm) using a 2-sample *t* test for a difference in mean pre/post change score gives the study a >85% probability of detecting a 1.5-point group difference on a 0‐10 rating scale (assuming an SD of 1.8). Conventional metrics suggest that a 30% change or difference score is clinically meaningful [98], and this sample size would provide adequate power to detect a group difference of approximately 20%. Most of the analyses in the proposed study will involve delta scores reflecting changes within participants (both uncorrected and adjusted least squares). We will examine the distribution of the delta scores between treatment arms and ascertain whether that distribution was normal for each of the study variables. Parametric (eg, analysis of covariance) or nonparametric analyses will be used on the variables based on whether the variables were numeric or categorical and showed normal distributions. Collectively, this sample size (N=100), together with our previously observed very high retention rates and the substantial efficacy of the platform, will provide more than adequate power to detect moderate-size effects. Participants will be randomized to the DT and TAU groups with equal allocation. The primary outcome measure will be the Patient Global Impression of Change (PGIC), which represents the participant’s overall belief about the efficacy of treatment on a 7-point categorical verbal rating scale. The scale ranges from (1) “no change or condition has gotten worse” to (7) “a great deal better and a considerable improvement that has made all the difference.” There will be prespecified secondary efficacy measures. Pain severity and pain interference with function will be evaluated with the Brief Pain Inventory. Neuropathic pain was assessed with the 7-item painDETECT questionnaire*.* Pain-related disability was evaluated with the Pain Disability Index. Psychological outcomes included the Hospital Anxiety and Depression Scale and the Pain Catastrophizing Scale. These outcome measures have been widely used in prior treatment trials, and their psychometric properties have been validated in US patients with chronic pain. All instruments were delivered via REDCap (Research Electronic Data Capture; Vanderbilt University). All efficacy measures, except for PGIC, will be taken at baseline and 3 months. PGIC will be assessed at 3 months.

The sample size calculation will be designed to confirm the hypothesis that the treatment assigned to the DT group will be associated with a greater PGIC score compared to the TAU control group at 3 months of 0.6 points with an SD of 1.0. The calculation assumes 85% power and a 2-sided type I error rate of 0.05. The estimated sample size of 100 will be increased to a target recruitment of 115 to account for 15% drop-out. The primary analysis of treatment effects will be conducted in the intention-to-treat (ITT) population, which will include all randomized participants. In addition, a prespecified subgroup analysis will be carried out. The study protocol predicts that participants in the DT group will demonstrate the greatest treatment effects.

The mean PGIC score at 3 months and the mean baseline to 3-month change scores for the secondary efficacy measures will be compared between the DT and TAU treatment groups by a mixed model for repeated measures (MMRM) analysis. In the presence of missing data, an MMRM analysis typically has more power than a 2-sample *t* test or analysis of covariance model. The ITT model will include fixed effects for treatment. A subgroup model will include all the parameters in the ITT model. This model will first be used to test for treatment heterogeneity by a significant interaction term at a 2-sided *P* value less than .15. A value of 1 (“no change”) will be assigned as the 3-month PGIC score if there is 3-month data for a participant. The outcome vector of the secondary efficacy measures will include a change score of zero for the initial (baseline) visit to account for participants with no treatment data. Missing covariates will be addressed with the missing-indicator approach. Correlations among measurements taken on the same participant will first be modeled with an unstructured covariance assumption. If the model fails to converge, then a first-order autoregressive covariance structure will be used. Marginal effects of treatment will be determined at 3 months, along with corresponding 2-sided *P* values. Comparisons will be deemed significant if the 2-sided *P* value is less than .05. Adjustments for multiple comparisons of secondary efficacy measures will be performed to account for the risk of elevated type I errors. Multiplicity corrections (eg, Bonferroni) will generally assume that outcomes are independent and will overcompensate for correlated measures, leading to increased type II errors. In this study, moderate correlations among many of the efficacy measures are expected. An MMRM analysis using all available data will impute missing data under a missing at random assumption. A sensitivity analysis will be performed using reference-based multiple imputation with the jump-to-reference method. The multiple imputation model will include the same covariates as the primary MMRM analysis. The imputed datasets will be analyzed with the original MMRM model and combined using the Rubin rule. The impact of missing data will be assessed by comparing the resulting estimates with the primary MMRM estimates.

Responder rates will be compared between treatment groups using logistic regression. Missing outcomes due to study withdrawal will be treated as nonresponders. The dependent variable in the model will be a binary variable indicating whether the participant was a responder or nonresponder. The model includes treatment assignment as an independent variable and baseline pain severity (Brief Pain Inventory average pain item) as a covariate. An interaction term between treatment and baseline pain sensitivity will be added for subgroup analyses of responder rates.

## Results

Training of the study personnel will begin in March 2026 after funding has been received and IRB approval. Recruitment of participants is expected to begin by June 2026. The recruitment phase of the study is expected to be completed in 6 months. The active phase of patient tracking is expected to last 1 year. Because participant enrollment will be staggered, the active phase of the study would be expected to last 18 months. It is expected that finalizing of study data and analysis, along with manuscript preparation, will begin 30 months from study initiation and last 6 months. Our hypothesis is that results will show that DT modeling using the biopsychosocial characteristics of each patient will be statistically significant, supporting the use of this approach for personalized medical care.

## Discussion

### Principal Findings

This paper offers a review of the literature about digital twin (DT) modeling and describes a study protocol for the use of DT methods to help persons with noncommunicable medical conditions of cardiovascular disease, type 2 diabetes, and chronic obstructive pulmonary disease. This study will use a smartphone app to monitor individuals with these conditions to help tailor treatment recommendations for each user. The HIPAA-compliant app will be supported by AI and computational analysis. Through this project, we intend to develop and validate a novel computational framework integrating AI-driven DT modeling to help personalize treatment.

While RCTs remain the gold standard for evidence-based medicine, clinicians treat heterogeneous patient populations whose disease presentation, treatment responses, and side effects vary greatly between individuals. Traditional RCTs rarely provide the multivariable data needed for evidence-based personalized medicine [56,99]. For clinicians, implementing personalized medicine remains complicated and time-consuming, and often these providers must make medical decisions without adequate support. Personalized medicine using DT methodology addresses individual variability by tailoring treatment to each person with the incorporation and benefit of genetic, physiological, biochemical, behavioral, and environmental data [24,55].

It is anticipated that this study will provide a methodology to validate the use of DTs for NCDs. NCDs are the leading cause of death and negative health-related impact in the world today. A body of evidence-based medicine has identified high-quality, effective treatment regimens that have contributed to internationally recognized treatment guidelines. Although evidence-based, authoritative, and widely accepted, the application of these guidelines has not yielded the expected results. Without a personalized medicine approach, application of these guidelines into clinical practice comes with significant shortcomings, as they rarely consider individual differences and the diversity of each individual, which are identified as biopsychosocial characteristics. Personalized medicine has been shown to help overcome these shortcomings. A key technology to deliver personalized medicine is the use of the DT.

We intend, as part of this proposed study, to develop a specific NCD DT Health Management App using AI and machine learning for the management of NCDs. Pilot data collection will include clinical measures and laboratory results, lifestyle and behavioral data, environmental factors, patient-reported outcomes, and wearable device metrics. Reflecting concerns about the lack of diversity found in evidence-based medicine, training of the algorithms needed to formulate the specific NCD DT will be monitored and corrected, as it is identified through a feedback loop incorporated within this study. Weekly feedback to users and providers will serve as input for decision-making and behavior change. We believe that this proposed preliminary study will offer insight into the development and implementation of DTs designed to reduce risk factors associated with the onset of NCDs. Ideally, the protocol described within this paper, incorporating a biopsychosocial approach, will help to provide the necessary data for future use in the development of AI algorithms. If the DT modeling is found to be effective and, as these algorithms are refined, personalized medicine using contextual messaging and predictive risk assessment will become a more viable treatment option to improve treatment outcomes and reduce health care use and expenditures.

A strength of this study resides in the ability of AI to construct a more complete DT of each unique patient. However, we anticipate several challenges with the conduct of this study. First, designing a scalable data architecture that securely integrates retrospective clinical data and real-time patient input will be an undertaking requiring input from many different sources. We plan to use a means for data extraction by integrating with the hospital medical record (HMR) system through the use of medical record numbers without jeopardizing security. We will incorporate the expertise of specialty clinicians, such as specialized medicine physicians, behavioral health clinicians, primary care providers, neuroscience researchers, IT technicians, and persons with lived experiences in dealing with NCDs. In preparation for this investigation, we have identified collaborators with the needed expertise to assist with this study.

Second, developing learning algorithms through the use of AI that update mechanistic parameters from longitudinal data will be challenging. Demonstrating predictive validity of the DT as it relates to real-world patient outcomes will be important to determine. We acknowledge a potential weakness of this study that may include unrecognized biases within an AI algorithm and its reliance on the current literature and published guidelines to generate treatment recommendations.

Finally, the study eligibility criteria have intentionally been designed to enable enrollment of participants resembling a real-life population of patients with NCDs. Result generalizability can be limited, however, by factors inherent to research that are not typical of clinical settings. Adherence to using the app, engaging in close follow-up with the study participants by the research personnel, and offering incentives for participation will be important. Studies have identified many patient factors that contribute to poor response to treatments. These include age, sensitivity to medications, emotional distress and catastrophizing, physical disability, early childhood trauma, history of substance use disorder, smoking cigarettes, and multiple medical comorbidities [84,96], which will be taken into consideration when evaluating use of the DT app. Also, some re-engineering to address security vulnerabilities and to integrate advanced AI capabilities for personalized predictive analytics may likely be required. The literature has noted the vital involvement of clinicians in eventually embracing the use of software in clinical practice [100-102]. We expect that the results of this trial will be useful in understanding how individuals who experience NCDs and those providers who treat them might incorporate the use of a DT app in daily practice. If the use of a DT app is found to be an effective treatment option for adults with NCDs, it will hopefully become a valuable tool to improve quality of life, increase function, reduce suffering, and address the large global burden of these conditions.

### Conclusions

NCDs are the leading cause of death in the world today. This is despite evidence-based, effective self-care and treatment options being widely available. NCDs are recognized as behavior-based diseases. We propose the use of a DT that will personalize care by including biopsychosocial characteristics in treatment plans, along with ongoing feedback to the patient that will lead to increased compliance with treatment recommendations and improvement in this high-impact public health crisis.

## Supplementary material

10.2196/75934 Multimedia Appendix 1 Communication methods and DT process elements for NCD care.

## Abbreviations

AI: artificial intelligence
API: application programming interface
DT: digital twin
EDITH: European Virtual Human Twin Initiative
EOC: episode of care
HIPAA: Health Insurance Portability and Accountability Act
IRB: Institutional Review Board
ITT: intention-to-treat
LLM: large language model
MMRM: mixed model for repeated measures
NCD: noncommunicable disease
PGIC: Patient Global Impression of Change
PLM: product lifecycle management
RCT: randomized controlled trial
REDCap: Research Electronic Data Capture
TAU: treatment as usual
