# Supplementary material for: Designing a Digital Twin for the Management of Noncommunicable Diseases: Protocol for a Pilot Study and Methodology Validation
Source: JMIR Res Protoc. 2026 Feb 9;15:e75934. doi: 10.2196/75934 (PMC12890778; doi:10.2196/75934)
Supplement: Multimedia Appendix 1 [file resprot-v15-e75934-s001.docx]

**Table S1.**Communication methods and APIs used in DT Program.

| **Legend** | **Data connection** | **API** |
| --- | --- | --- |
|  | Blue Tooth | REST (Representational State Transfer) |
|  | Cellular Network | HTTPS (Hypertext Transfer Protocol Secure) |
|  | Cloud Based DT | PaaS (Platform as a Service) |

**Table S2.** Detail of DT process elements and steps for NCD care.

| **Patient specific digital twin** | |
| --- | --- |
| Initial Inputs | |
|  | Clinical care begins with an assessment of the patient’s present status including symptoms if any, current laboratory testing, past medical history, including review of active problem list, determination of risk factors, health care barriers if any, family history, previous treatments, beliefs, ethnic and cultural background, psychological traits, physical exam and functional status. Taken together a patient profile can be developed and distilled into three domains that include:   1. Medical diagnosis that requires an intervention and treatment. 2. Psychological traits that can interfere with optimal care 3. Physical functioning that can present barriers to treatment, self-care and improvement.   All questionnaires are available through a secure mobile app or internet portal. |
|  | This step identifies, reviews for missing data points and ensures compatibility with DT algorithm. Coding of problem list is included in this step. |
|  | Clinical assessment of the patient is completed at this step and a primary diagnosis is formulated |
|  | All data is stored for review and retrieval throughout the episode of care in real time. |
|  | Primary and secondary diagnoses are coded and evidence-based treatment algorithms are retrieved. |
| Formulate Treatment Plan | |
|  | DT constructed based on patient evaluation including patient risk factors. Treatment modeling including:   1. Medical diagnosis and treatment. 2. Psychological traits and treatment recommendations if needed. 3. Physical functioning with treatment recommendations if needed. |
|  | DT developed using clinical data obtained at initial evaluation |
|  | Treatment plans from DT and provider compared. |
|  | Patient response to treatment recorded |
|  | Continuous data collection used to refine DT profile |
| Active Treatment Phase | |
|  | Treatment response of patient compared to DT expected response |
|  | Data reviewed from patient tracking, used to update DT |
|  | Treatment next steps from provider and DT compared. |
| End of Episode of Care | |
|  | Treatment concluded |
|  | Data analysis and refinement of DT care pathway |
